# Unbridled Threat of Gas Gangrene in a Patient With Uncontrolled Diabetes Mellitus: A Compelling Case Report of Clostridium perfringens Infection

**DOI:** 10.7759/cureus.50614

**Published:** 2023-12-16

**Authors:** José Luis Serafio-Gómez, Melanie Bustillos-Ponce, Diego Emmanuel Almeida-Muñoz, Joel Armando Parra-Hernández, Julio César Pompa-Díaz, José F. de la Torre-Ramos

**Affiliations:** 1 General Surgery, Chihuahua City General Hospital “Dr. Salvador Zubirán Anchondo”, Chihuahua, MEX; 2 General Surgery, Hospital General de Hidalgo del Parral, Chihuahua, MEX; 3 General Surgery, Chihuahua City General Hospital "Dr. Salvador Zubirán Anchondo", Chihuahua, MEX

**Keywords:** wound management, anaerobic infection, diabetes mellitus, gas gangrene, clostridium perfringens

## Abstract

*Clostridium perfringens*, a Gram-positive anaerobic bacterium, is well-known for its association with gas gangrene, a severe and rapidly progressing infection characterized by tissue gas production and necrosis. In this case report, we present the instance of a 64-year-old male with poorly controlled diabetes mellitus who developed a *C. perfringens*-related infection following a traumatic foot wound. The report emphasizes the critical significance of early diagnosis and aggressive treatment in *C. perfringens* infections, particularly in patients with underlying risk factors. Detailed accounts of clinical findings, laboratory results, computed tomography, and surgical interventions are provided. A multidisciplinary approach proved essential for successful management. The inherent scholarly value of this case is substantiated by its meticulous documentation of the clinical trajectory, diagnostic modalities, and treatment modalities employed. The intricate collaboration across diverse medical disciplines, the uncommon manifestation of the infection following a traumatic foot wound, and the favorable outcome achieved through prompt and multidisciplinary intervention collectively contribute to the exceptional nature and didactic significance of this case. The dissemination of such clinical experiences assumes paramount importance in advancing medical scholarship, cultivating awareness, and engendering a profound comprehension of the complexities associated with *C. perfringens* infections, thereby enriching the wider scientific and medical community.

## Introduction

*Clostridium perfringens*, a Gram-positive anaerobic bacterium, is ubiquitously distributed in the environment and is commonly found in the human gastrointestinal tract. Renowned for its proclivity to induce severe infections, particularly in wounds contaminated with its spores or in necrotic tissues, this bacterium is notably associated with gas gangrene: a potentially life-threatening condition characterized by the production of gas within infected tissues and rapid tissue degradation [1].

*C. perfringens* type A is the major cause of traumatic gas gangrene, although other histotoxic clostridia may also be responsible for this sporadic, but fulminant and often fatal, disease. The major toxin produced by gas gangrene strains of *C. perfringens* is α-toxin, a zinc metallophospholipase that has both phospholipase C and sphingomyelinase activity α-toxin was the first bacterial toxin to be shown to have enzymatic activity. Two independent studies using different experimental approaches have shown that α-toxin is essential for *C. perfringens*-mediated myonecrosis. First, immunization studies in mice using recombinant α-toxin variants purified from *Escherichia coli*, and are therefore devoid of any other *C. perfringens* toxins, showed that the C-terminal domain of α-toxin was immunoprotective. Second, mutation of the α-toxin structural gene (plc or cpa) abrogated the ability of the bacterium to cause clostridial myonecrosis in a murine model. Virulence was restored by complementation in trans with a recombinant plasmid containing the wild-type plc gene, thereby fulfilling molecular Koch’s postulates and providing proof of the essential role of this toxin in disease [2].

Gas gangrene, classified into three types (posttraumatic, postoperative, and spontaneous) presents distinctive clinical profiles. Traumatic gangrene, the pathogenesis of these infections, involves three distinct stages, regardless of whether the source of infection involves a traumatic wound. Stage 1 involves a disruption of the blood supply, causing the redox potential in tissues to drop to a level that facilitates the germination of spores and/or the growth of infecting clostridial cells. Stage 2 involves bacterial growth and the establishment of conditions for toxin production. In* C. perfringens*, the regulation of toxin production primarily utilizes the two-component signal transduction system VirSR and a quorum-sensing system akin to an accessory growth regulator, along with other regulatory networks. Stage 3 encompasses cellular and tissue damage mediated by toxins, leading to necrosis, systemic toxicity, and clinical disease. Historically linked to war-related injuries and natural disasters, remains a significant cause, while spontaneous gas gangrene is particularly prevalent among immunosuppressed individuals, encompassing those with diabetes, tumors, undergoing chemotherapy, or with a history of drug abuse [2]. Notably, deep penetrating injuries contribute to approximately 70% of gas gangrene cases [3]. This malady instigates a cascading series of events, including toxin production, leading to accelerated tissue destruction, thereby posing a lethal threat [1].

*C. perfringens* produces an array of toxins, with alpha toxin being the most relevant in the pathogenesis of gas gangrene. This potent toxin assumes a pivotal role in tissue degradation and the facilitation of gas production within the infected milieu [3-5]. Clinical presentations of gas gangrene typically involve signs of infection, such as fever, pain, inflammation, and wound discharge characterized as dishwater-looking with a musty odor. Vascular involvement, supplying extensive regions of infected tissue, results in the necrosis of subcutaneous fat, fascia, and deeper muscular structures. Nerve damage may mitigate expected pain levels [6,7].

For the comprehensive evaluation of individuals suspected to have gas gangrene, a thorough assessment should encompass a complete blood count (CBC) and comprehensive metabolic panel (CMP) along with targeted diagnostic procedures such as imaging studies and bacterial cultures. Early diagnosis and intervention are paramount, given the fulminant nature of gas gangrene [8-12].

In conclusion, the multifaceted nature of gas gangrene, particularly in the context of *C. perfringens* infections, underscores the importance of a nuanced understanding of its clinical presentations, risk factors, and the necessity for prompt, multidisciplinary management. This comprehensive perspective is essential not only for the effective treatment of individual cases but also for advancing our broader understanding of the pathophysiology and therapeutic strategies associated with this potentially devastating condition.

## Case presentation

**Figure 1:**
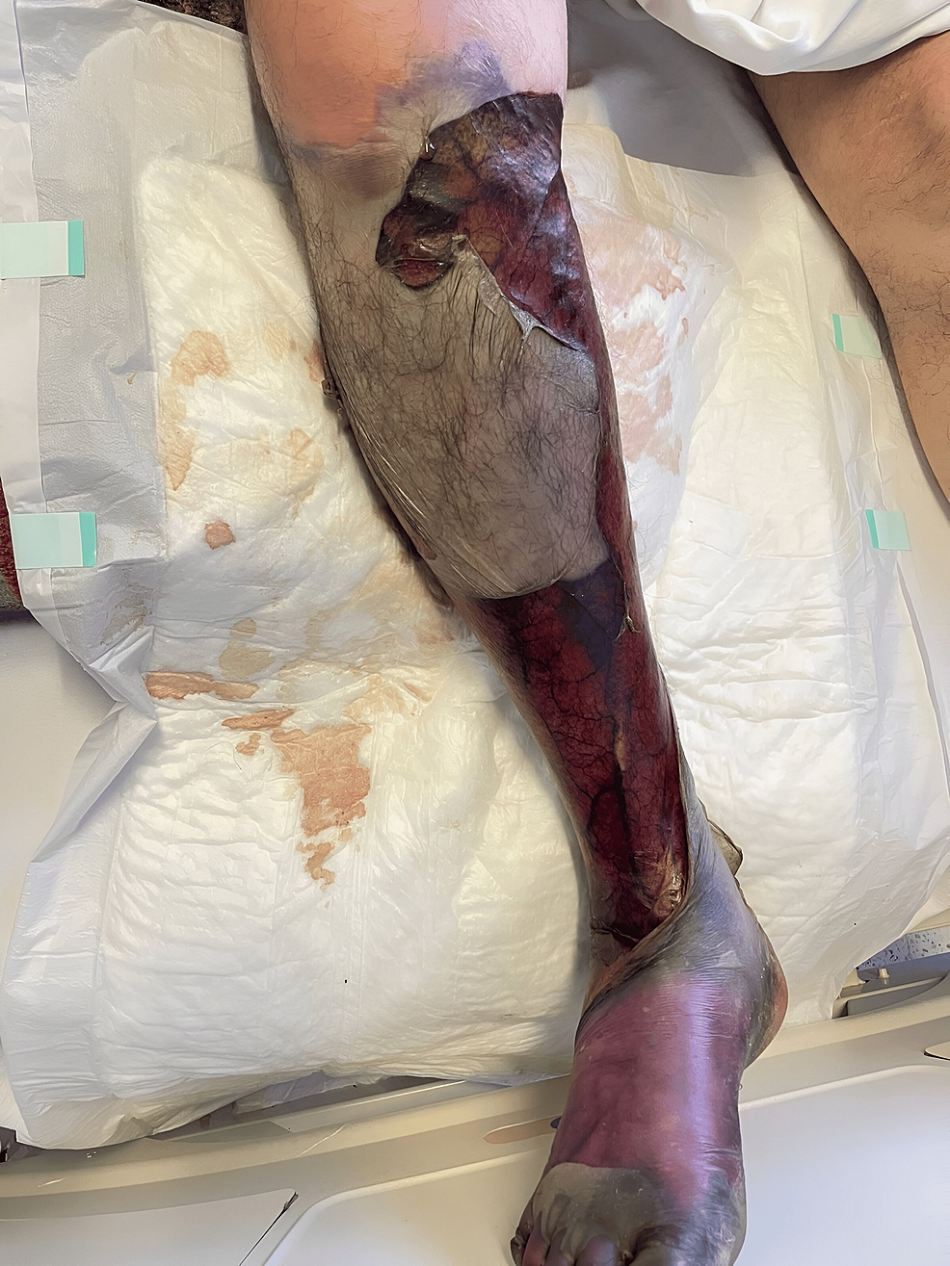
Visual Manifestation of Gas Gangrene Progression The skin shows noticeable discoloration, ranging from deep purples and blues to mottled hues, signaling compromised blood circulation. Darkened patches indicate distinct areas of tissue necrosis, where the infection has taken its toll. The skin, once a protective barrier, now reflects the relentless progression of gas gangrene.

A 64-year-old gentleman with a history of poorly controlled diabetes mellitus commenced experiencing symptoms three days antecedent to his presentation at the emergency department. He reported an incident wherein he inadvertently stepped on a rusted nail with his right foot, subsequently manifesting pain, edema, blistering, and necrosis, initially localized to the foot and progressing proximally to involve the right knee (Figure 1).

During the physical examination, the patient exhibited the following vital sign parameters: blood pressure of 130/70 mmHg, heart rate of 100 beats per minute, respiratory rate of 28 per minute, temperature of 40 degrees Celsius, and glucometry of 200 mg/dL.

The physical examination revealed in the right lower extremity the presence of pain upon mobilization across all ranges of motion, accompanied by blistering and telangiectasias. Subcutaneous emphysema was discerned, extending from the foot to the right inguinal region, concomitant with crepitus upon palpation. An incision with mild purulent discharge was identified at the heel, while the remainder of the examination demonstrated no aberrations.

Upon admission to the emergency facility, the patient expeditiously underwent a multidisciplinary treatment regimen, initiating dual antibiotic therapy following the IDSA guidelines (vancomycin + piperacillin). Additionally, the management of the comorbidity of diabetes mellitus entailed a dual insulin regimen. Intermediate-acting NPH insulin, calculated on a per-kilogram basis, was administered in a thrice-daily regimen, alongside rapid-acting insulin for rescue purposes. After these interventions, comprehensive laboratory analyses were conducted, and the ensuing table delineates the results of the hematological assessment (Table 1).

**Table 1:** Reference laboratory values in comparison with the values found in the patient. The reference range of values is based on the laboratory providing to the hospital.

Laboratory	Reference range*	Patient value
Glucose	74-106 mg/dL	476 mg/dL
Creatinine	0.5-1 mg/dL	2.2 mg/dL
Chloride	98-111 mmol/L	97 mmol/L
Phosphorus	2.5-4.5 mg/dL	3.1 mg/dL
Potassium	3.5-5.1 mmol/L	4.40 mmol/L
Sodium	137-145 mmol/L	135 mmol/L
Calcium	8-10mg/dL	9 mg/dL
Leukocytes	4.5-11 K/uL	18.7 mg/dL
Neutrophils	1.8-7 K/uL	16.71 K/uL
Hemoglobin	13- 17 g/dL	16.70 g/dL
Hematocrit	42-50%	51.2%
Platelets	150-450k uL	169k/uL
Prothrombin time	11-14 sec	22.3 sec
INR	1	1.7
Activated partial thromboplastin time	20-45 sec	35.9 sec

**Figure 2:**
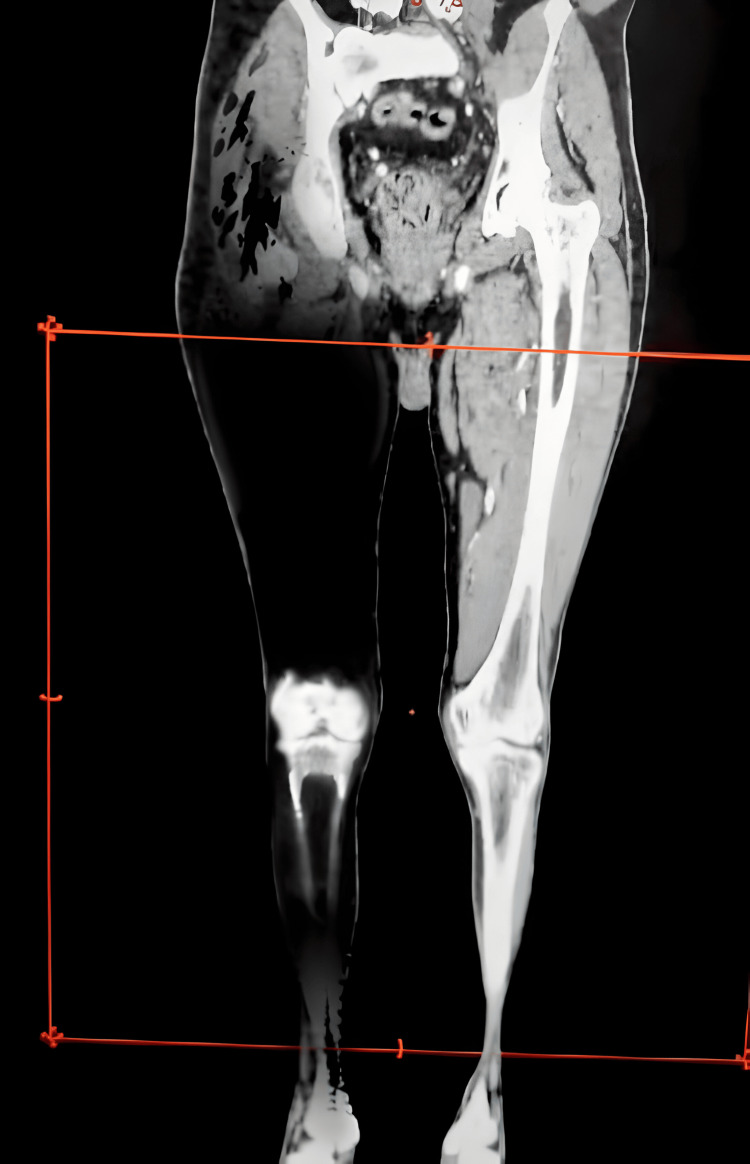
Tomography Assessment; Profound Tissue Destruction The tomography scan of the affected pelvic limb reveals extensive necrosis, with a notable absence of appreciable tissue. This finding indicates severe cellular damage and necrotic tissue loss. The darkened areas in the image signify regions where tissue destruction has occurred, offering a visual representation of the extent of necrosis.

**Figure 3:**
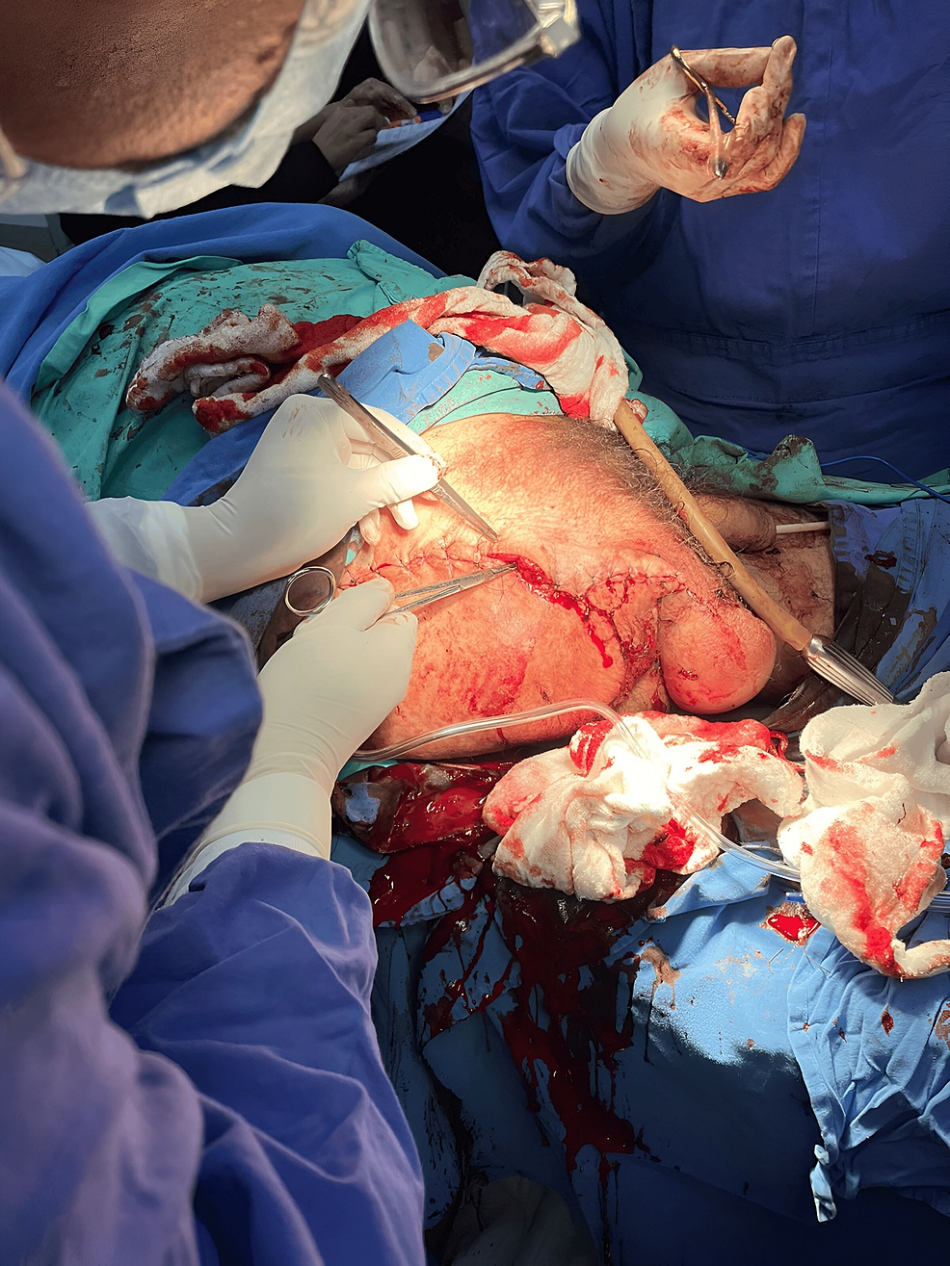
Surgical Management: Hip Disarticulation The decision to proceed with a hip disarticulation was dictated by the distinctive characteristics of the case, specifically, gas gangrene. This illustration encapsulates the comprehensive nature of the surgical procedure, demonstrating the excision of the entire lower extremity to address significant tissue damage and curtail the advancement of the gas gangrene infection. The selection of hip disarticulation is emphasized by the imperative to ensure effective control of infections and uphold the overall health of the patient.

The gravity of tissue damage in the affected region is vividly depicted in Figure 3, wherein a tomographic examination reveals extensive necrosis and a conspicuous absence of appreciable tissue, coupled with the lack of vascular structures in the angiotomographic rendition. This imagery encapsulates the profound impact of the pathological condition on the anatomical framework, underscoring the imperative for expeditious intervention and the critical necessity for meticulously tailored therapeutic measures. The areas of diminished radiodensity in the scan delineate regions where substantive tissue destruction has ensued, providing a visual representation of the magnitude of necrosis, spanning the entirety of both muscular and vascular components (Figure 2).

Upon discerning the presence of gas diffused throughout the entire limb, extensive damage to both the muscular and vascular bundles in the tomographic assessment, and recognizing the potential for precipitous deterioration of the pathology, a judicious determination was reached to expeditiously proceed with a hip disarticulation on the day of admission. This decision underwent evaluation by the anesthesiology service and expeditiously transitioned from the emergency room to the operating theater. Throughout the surgical intervention, a substantial accumulation of gas, emblematic of gas gangrene, was identified within the affected region, extending proximally up to the second third of the thigh, impacting all anatomical bundles: muscular, vascular, and nervous alike. The predetermined course of action persevered as planned, notwithstanding the identification of gas in the inguinal region on tomography, which, upon closer inspection, pertained solely to soft tissues and warranted no additional procedural intervention.

The procedural conclusion unfolded devoid of complications, characterized by the meticulous execution of flaps and unobtrusive wound closure, concomitant with the implementation of active drainage (Drenovac).

The judicious choice to effectuate a hip disarticulation rested upon clinical assessment, wherein the pervading gas presence posed a substantial risk of exacerbating the patient's clinical state. Given the gravity of gas gangrene and the imminent prospect of rapid degeneration, the surgical team opted for a hip disarticulation to ensure the thorough extirpation of the compromised tissue and the circumscription of the infectious process. This strategic approach aimed at abating the progressive nature of gas accumulation and muscular necrosis, as ascertained during the surgical intervention, thereby mitigating the risk of concomitant complications and fostering efficacious treatment. The decision to pursue hip disarticulation was meticulously weighed against the backdrop of the observed pathology, underscoring the imperativeness of arresting the advancement of gas gangrene and forestalling systemic complications (Figure 3).

After the surgical intervention, the patient demonstrated conspicuous amelioration, concomitant with concurrent antibiotic therapy, characterized by a reduction in inflammatory and infectious mediators. Notably, the exigency for blood transfusion did not manifest, and the patient exhibited a favorable clinical trajectory without exacerbation.

However, on the sixth day postoperatively, the patient manifested purulent discharge from the wound, precipitating the removal of lateral sutures. Subsequent cultivation reported on the third day revealed positivity for *E. coli*, with sensitivity to vancomycin. Consequently, a judicious resolution was made to persist with the identical antibiotic regimen, supplemented by a regimen of daily wound care employing povidone-iodine and saline solution. Following the management of the stipulated protocol for seven days, marked by satisfactory progression and the development of granulation tissue, suture reinstatement was performed, which culminated in the patient's discharge.

## Discussion

This case report underscores the critical significance of early diagnosis and proactive treatment in instances of *C. perfringens* infections. The surgical procedure, involving hip disarticulation, was promptly conducted on the same day the patient entered the emergency room, reflecting the urgent need for intervention. However, a three-day delay in initiating specific treatment measures ensued, primarily attributed to the time required for processing and reporting the culture results. This period was crucial for obtaining a comprehensive understanding of the microbial profile and guiding the formulation of targeted postsurgical therapeutic strategies. The immediate surgical response underscored the critical nature of the patient's condition, while the subsequent delay was a deliberate and necessary step to ensure an effective approach based on the identified pathogens.

The pathogenesis of these infections follows a trajectory characterized by three delineated stages, irrespective of whether the source of infection involves a traumatic wound. In the initial stage, there is a disruption in the blood supply, precipitating a reduction in the redox potential within tissues, thereby creating an environment conducive to the germination of spores and/or the proliferation of infecting clostridial cells. The second stage is distinguished by bacterial proliferation and the establishment of conditions favorable for toxin production. In the case of *C. perfringens*, the regulation of toxin production predominantly involves the two-component signal transduction system VirSR and a quorum-sensing system analogous to an accessory growth regulator, in conjunction with other regulatory networks. The third stage encompasses cellular and tissue damage mediated by toxins, culminating in necrosis, systemic toxicity, and clinical manifestations of the disease. In this particular case, pathological confirmation through cultures underscores the paramount significance of timely intervention. It is imperative to underscore that mortality in the absence of treatment is absolute at 100%, and in pertinent and intricate cases, notwithstanding treatment, mortality may ascend to 40%. This emphasizes the compelling necessity for expeditious and efficacious measures in addressing these infections to ameliorate clinical outcomes and mitigate associated morbidity.

The management of the patient's case reflected a collaborative approach that drew on the expertise of multiple medical specialties. The surgical intervention, led by the General Surgery team, involved close coordination with infectious disease specialists, microbiologists, and radiologists. The prompt surgical response was complemented by the diligent monitoring of laboratory results and imaging studies. This collaborative and comprehensive approach, involving specialists from diverse fields, ensured a well-rounded and effective management plan for the patient.

Swift identification of risk factors, notably poorly controlled diabetes mellitus, coupled with the implementation of tailored surgical and antibiotic interventions, proves paramount for the successful management of this potentially life-threatening infection. The findings emphasize the importance of a multidisciplinary approach and timely intervention in ensuring positive patient outcomes in the face of such challenging clinical scenarios.

The critical role of the alpha toxin produced by *C. perfringens* in the pathogenesis of gas gangrene underscores the imperative for early and aggressive intervention. The accelerated progression of the infection mandated surgical intervention, in this case coxofemoral disarticulation, to effectively control the infection and avert further complications. The choice of coxofemoral disarticulation as the surgical intervention was justified by several factors. Given the severe nature of the gas gangrene associated with *C. perfringens* infections and the rapid progression of tissue destruction, a more radical approach was deemed necessary. Coxofemoral disarticulation, involving the removal of the entire hip joint and proximal femur, allowed for extensive debridement of the infected tissue, minimizing the risk of residual infection and promoting optimal wound healing. Additionally, the procedure provided a more effective means of controlling the spread of gas and toxins associated with the infection. The decision for coxofemoral disarticulation was further supported by its success in achieving the desired outcomes, highlighting the appropriateness of this targeted surgical measure in the management of such complex cases [3-5].

In summary, the synthesis of clinical experience and scientific evidence underscores the paramount importance of providing comprehensive and timely care-from the initial diagnosis to treatment-to ensure optimal outcomes. This integrated approach is pivotal in navigating the complexities of these infections, emphasizing the need for a meticulous and integrated healthcare strategy to achieve the best possible patient outcomes.

The suspicion of *C. perfringens* infection was rooted in the patient's clinical presentation and the circumstances surrounding the traumatic foot wound. With a history of poorly controlled diabetes mellitus, the emergence of severe symptoms-including rapid tissue necrosis, subcutaneous emphysema, and palpable crepitus-raised significant concerns about gas gangrene. Recognized as a well-established causative agent in compromised wounds with compromised blood supply, *C. perfringens* became a focal point of investigation. The swift onset and progression of symptoms, coupled with characteristic clinical findings, triggered a robust suspicion of *C. perfringens* infection. Consequently, diagnostic measures, including laboratory tests and wound cultures, were promptly initiated to confirm and guide the subsequent course of treatment [9-12].

## Conclusions

This case report underscores the utmost significance of early diagnosis and efficacious treatment in cases involving *C. perfringens* infections. The expeditious identification of risk factors, notably poorly controlled diabetes mellitus, coupled with the implementation of judicious surgical and antibiotic strategies, assumes critical importance for the successful mitigation of this potentially lethal infection. Nevertheless, it is imperative to acknowledge the encountered challenges during the patient's posttreatment monitoring, given the considerable geographical distances separating the patient's residence-situated four hours away from the healthcare facility where treatment was administered for the aforementioned pathology. These geographical disparities pose substantial impediments to accessibility and the seamless continuation of medical care, underscoring the exigency to address logistical challenges endemic to healthcare provision.

The pivotal role played by the alpha toxin produced by *C. perfringens *in the pathogenesis of gas gangrene accentuates the imperative for early and assertive intervention in instances of such a nature. In this specific case, the decision to undertake hip disarticulation proved indispensable for the effective containment of the infection and the preclusion of additional complications. The selection of this precise surgical intervention was predicated upon a scrupulous assessment of the extent of tissue damage and the imperative for resolute action to ensure the comprehensive eradication of the pathogenic agent. In summary, the confluence of clinical acumen and empirical evidence vehemently advocates for a comprehensive and timely care paradigm, spanning from the initial diagnostic phase to the therapeutic intervention, thereby ensuring optimal outcomes for patients grappling with infections caused by *C. perfringens*. Additionally, the nuanced complexities of this particular case underscore the critical importance of surmounting logistical impediments in healthcare delivery, especially in instances necessitating prolonged patient follow-up over extensive distances, thereby contributing substantively to the ongoing enhancement of healthcare quality.

## References

[1] Takazawa T, Ohta J, Horiuchi T, Hinohara H, Kunimoto F, Saito S (2016). A case of acute onset postoperative gas gangrene caused by Clostridium perfringens. BMC Res Notes.

[2] Nagahama M, Takehara M, Rood JI (2019). Histotoxic clostridial infections. Microbiology spectrum.

[3] Stevens DL, Bryant AE (2017). Necrotizing soft-tissue infections. N Engl J Med.

[4] Mehdizadeh Gohari I, A Navarro M, Li J, Shrestha A, Uzal F, A McClane B (2021). Pathogenicity and virulence of Clostridium perfringens. Virulence.

[5] Stevens DL, Aldape MJ, Bryant AE (2012). Life-threatening clostridial infections. Anaerobe.

[6] Roberts EJ, Martucci JA, Wu D (2018). The unusual presence of gas from a puncture wound: a case report. J Foot Ankle Surg.

[7] Bryant AE, Stevens DL (2010). Clostridial myonecrosis: new insights in pathogenesis and management. Curr Infect Dis Rep.

[8] Sarvari KP, Vasas B, Kiss I (2016). Fatal Clostridium perfringens sepsis due to emphysematous gastritis and literature review. Anaerobe.

[9] Devaney B, Frawley G, Frawley L, Pilcher DV (2015). Necrotising soft tissue infections: the effect of hyperbaric oxygen on mortality. Anaesth Intensive Care.

[10] Finsterer J, Hess B (2007). Neuromuscular and central nervous system manifestations of Clostridium perfringens infections. Infection.

[11] Nichols RL, Smith JW (1994). Anaerobes from a surgical perspective. Clin Infect Dis.

[12] Yang Z, Hu J, Qu Y, Sun F, Leng X, Li H, Zhan S (2015). Interventions for treating gas gangrene. Cochrane Database Syst Rev.

