# Correction: Unbridled Threat of Gas Gangrene in a Patient With Uncontrolled Diabetes Mellitus: A Compelling Case Report of Clostridium perfringens Infection

**DOI:** 10.7759/cureus.c178

**Published:** 2024-05-24

**Authors:** José Luis Serafio-Gómez, Melanie Bustillos-Ponce, Diego Emmanuel Almeida-Muñoz, Joel Armando Parra-Hernández, Julio César Pompa-Díaz, José F. de la Torre-Ramos

**Affiliations:** 1 General Surgery, Chihuahua City General Hospital “Dr. Salvador Zubirán Anchondo”, Chihuahua, MEX; 2 General Surgery, Hospital General de Hidalgo del Parral, Chihuahua, MEX; 3 General Surgery, Chihuahua City General Hospital "Dr. Salvador Zubirán Anchondo", Chihuahua, MEX

The authors list of this article has been corrected to include senior author José F. de la Torre-Ramos, who was mistakenly omitted from the original author list. Dr. de la Torre-Ramos has been added as the last and corresponding author. The authors greatly regret that this omission was not identified and corrected prior to publication.

